# Obstructive Sleep Apnea: A Potential Sleeping Giant of Sport Performance and Health?

**DOI:** 10.1007/s40279-026-02427-2

**Published:** 2026-05-19

**Authors:** Matthew Driller, Alan J. Pearce, Nicole Lovato

**Affiliations:** 1 School of Allied Health, Human Services, and Sport, La Trobe University, Melbourne, Australia; 2 Swinburne Neuroimaging Facility (SNI), School of Health Sciences, Swinburne University of Technology, Melbourne, Australia; 3 Flinders Health and Medical Research Institute: Sleep Health, College of Medicine and Public Health, Flinders University, Adelaide, Australia

Obstructive sleep apnea (OSA) is a common sleep-related breathing disorder in which the upper airway repeatedly narrows or collapses during sleep, causing pauses or reductions in breathing, oxygen desaturation and fragmented sleep. Clinically, it is diagnosed using specialized tools such as polysomnography, when the apnea–hypopnea index (AHI) is ≥ 5 events·h^−1^, in the context of compatible symptoms [1]. In the general adult population, OSA (AHI ≥ 5 events·h^−1^) is now estimated to affect roughly 10–30% of adults, with moderate-to-severe disease (AHI ≥ 15 events·h^−1^) present in 6–17% [2, 3]. Prevalence is consistently higher in men than women and escalates with age and adiposity. In cohorts with BMI ≥ 30 kg·m^2^, approximately 40–70% of individuals meet criteria for at least mild OSA (i.e. AHI ≥ 5 events·h^−1^) [4, 5]. Emerging evidence suggests that OSA may be a “sleeping giant” in several sporting contexts: prevalent in certain athlete groups (especially those in contact/collision-based sports), potentially performance-limiting and health-threatening, and largely invisible to routine screening and monitoring.

Elite athletes are already recognized as vulnerable to inadequate and irregular sleep, driven by late-night training and/or competition, travel, stress, and congested schedules [6]. Original studies, narrative reviews and consensus statements show that elite athletes frequently obtain < 7 h sleep per night and report poor sleep quality, with consequent impairments in mood, cognitive function, metabolism, and recovery that are relevant to training adaptation and performance [6–9]. However, most of this work has focused on behaviorally driven sleep restriction and insomnia-like complaints, rather than sleep-disordered breathing. The assumption that OSA is confined to older, sedentary, obese adults is increasingly untenable.

Objective sleep-study data in athletes, although still limited, suggest that OSA is not rare in contact and collision sports. In an elite rugby union team, Dunican et al. [10] found that 24% of players had OSA (AHI ≥ 5 events·h^−1^). In professional rugby league, Caia et al. [11] reported an even higher prevalence: 10 of 22 players (46%) were diagnosed with either mild or moderate OSA using home-based polysomnography, with forwards and Polynesian athletes overrepresented among cases. Extending beyond single-team data, Howarth et al. [12] synthesized 14 studies across American football, rugby union, rugby league, ice hockey, and judo, reporting a pooled OSA prevalence of 30% (95% confidence intervals (CI) 24–36%) in contact-sport athletes, exceeding general-population estimates. Notably, elevated prevalence was observed both in current and retired athletes, and in several college American football cohorts, more than half of players met OSA criteria [12].

Subjective symptom and screening data also point to a meaningful pool of athletes who may warrant further assessment. In 175 elite or highly trained rugby sevens, rugby union and cricket athletes, Swinbourne et al. [13] found that 50% met criteria for “poor sleep” (Pittsburgh Sleep Quality Index > 5), 28% had clinically significant daytime sleepiness, and 38% reported habitual snoring; 8% reported witnessed apneas. Although questionnaire data alone cannot diagnose OSA, these findings show that classical risk features and symptoms are not uncommon in athletes, particularly those with greater body mass and neck circumference, and can be used to prioritize referral for objective testing.

Risk factors placing athletes at a higher likelihood of having OSA may include increased body mass and central adiposity (e.g., collision sports, throwers, rowers, heavyweight combat sports), unfavorable craniofacial anatomy, a tendency to sleep supine, and exposure to repeated head impacts or prior traumatic brain injury/concussion [14]. In addition, nonmodifiable factors such as male sex and advancing age and belonging to higher-risk ethnic groups (e.g., Pacific Islander, Māori, African American, Hispanic, and several Asian populations) appear to confer greater OSA prevalence and/or severity [15–19], likely mediated by greater neck circumference and craniofacial morphology. However, emerging research demonstrates that OSA can also arise from nonanatomical traits such as low arousal threshold, high loop gain, and impaired upper airway muscle responsiveness [20]. These mechanisms highlight that body habitus alone is not a reliable predictor of risk, and physiologically informed screening approaches are needed in athletes. Practically, this can be implemented using a stepped approach within existing sport medicine frameworks (e.g., preseason medicals, annual health checks, or concussion follow-up): brief symptom/risk screening to flag athletes for referral, followed by targeted home sleep testing or laboratory polysomnography when indicated.

From a mechanistic standpoint, OSA presents several plausible pathways through which it may impair performance, recovery, and wellbeing. Sleep fragmentation and poorer overall sleep quality are common in OSA and are associated with excessive daytime sleepiness, impaired vigilance, slower reaction time, reduced executive function, and mood disturbance [6]. However, these manifestations are heterogeneous: several individuals (e.g., those with a higher arousal threshold) may experience fewer overt awakenings despite significant respiratory events, whereas others have prominent fragmentation [20]. Autonomic dysregulation and surges in sympathetic activity may also compromise cardiovascular recovery, glycemic control and anabolic–catabolic balance, counteracting the desired adaptations to training [21–23]. If OSA is accompanied by reduced or fragmented N3 (deep/slow-wave) sleep, this may blunt sleep-associated growth hormone pulses. Given that ∼ 60–70% of daily growth hormone secretion occurs during early slow-wave sleep, reductions may impair muscle repair and recovery [24–27]. In collegiate athletes, Grandner and colleagues showed that OSA symptoms were independently associated with higher stress and depressive symptoms and lower perceived social support, even after accounting for other sleep problems [28], suggesting that OSA-related symptomatology may undermine both psychological wellbeing and the social resources that buffer training and injury stressors. “Sleep hygiene” interventions alone are unlikely to normalize these psychophysiological disruptions if the underlying issue is unrecognized OSA. Notably, OSA frequently co-occurs with insomnia (often termed COMISA), with studies and reviews suggesting that up to ~ 40–60% of patients with OSA report clinically significant insomnia symptoms, and ~ 30–40% of patients with insomnia meet OSA criteria [29, 30]. Athletes may therefore present with overlapping complaints such as difficulty initiating sleep, nocturnal awakenings, and daytime fatigue, reinforcing the need for integrated sleep assessment and management that distinguish behavioral sleep restriction, insomnia, and sleep-disordered breathing.

The intersection between OSA and sport-related concussion (SRC) is particularly concerning for athlete health and career longevity. Contact-sport athletes already face elevated SRC risk, and Howarth et al. [12] explicitly highlight head injuries as a potential contributor to the high OSA burden observed in contact sports. It is plausible that untreated OSA, via excessive sleepiness, impaired vigilance, or slowed reaction time, could contribute to injury risk, but direct evidence linking OSA to SRC incidence in athletes is currently lacking and should be framed as hypothesis-generating. Conversely, traumatic brain injury (TBI), including SRC, can itself precipitate or exacerbate sleep-disordered breathing via damage to brainstem respiratory control, altered muscle tone or anatomical changes [31]. Reviews of sleep after TBI consistently report high rates of sleep disorders, with OSA one of the most common, and suggest that OSA may be both a risk factor for, and a consequence of, TBI [31].

Sleep disturbances more broadly are robust predictors of persistent postconcussion symptoms, with persistent sleep problems associated with slower clinical recovery, ongoing cognitive deficits, and mood disturbance [14, 31]. If an athlete with SRC also has undiagnosed OSA, nocturnal hypoxemia and sleep fragmentation may further impair neuroplasticity, glymphatic clearance, and the restorative processes necessary for brain recovery [32]. In practice, this may present as the “complex” concussion case that does not respond to standard graded return-to-sport protocols or as recurrent minor concussions against a backdrop of chronic fatigue and inattention. However, OSA is rarely considered systematically in postconcussion pathways in sport.

What should sports medicine and performance teams do with this information? The recent Amsterdam 2022 International Consensus Statement on Concussion in Sport provides broad guidance on SRC prevention, assessment, and management and highlights sleep disturbance as a common postconcussion symptom, but it does not specifically recommend routine screening for primary sleep disorders such as OSA within concussion pathways [33]. In contrast, consensus recommendations from the NCAA Interassociation Task Force advocate incorporating sleep-disorder screening into preparticipation evaluations, underscoring that conditions such as OSA should be considered part of routine athlete health surveillance rather than niche sleep-medicine concerns [34]. Clinicians should explicitly consider OSA when athletes present with nonrestorative sleep, loud snoring, nocturnal choking, morning headaches, or disproportionate daytime sleepiness, particularly in heavier, larger-necked or contact/collision-sport athletes [10–12]. Consumer wearables are not diagnostic tools for OSA; however, several devices and contactless sensors show promise for screening/flagging and risk-stratification when integrated into an appropriate clinical pathway (e.g., prompting referral rather than “diagnosing” OSA) [35]. Brief questionnaires such as STOP-Bang or Berlin can stratify risk, but they have not been robustly validated across diverse athletic populations; available work (e.g., in American football) suggests potential utility but uncertain generalizability beyond the specific cohorts studied [36]. Accordingly, the gold standard for diagnosis remains polysomnography or high-quality home sleep studies, and athlete-specific validation and adaptation of screening instruments are urgently required to avoid the underrecognition of OSA in younger, leaner, or physiologically distinct cohorts. Identification of suspected OSA in sport settings must trigger a genuinely multidisciplinary response.

Our recent work [37] outlines a collaborative framework for managing athletes with sleep problems, emphasizing a clear role delineation between sports physicians, sleep physicians, physiologists, psychologists, coaches, and performance staff. Within such a model, brief, validated tools such as the Athlete Sleep Screening Questionnaire (ASSQ) can act as a front-line clinical screen to identify athletes with significant sleep complaints and “red flags” for OSA (e.g., loud snoring, witnessed apneas, unrefreshing sleep) and to trigger timely referral for formal sleep assessment [38]. Importantly, acceptability is a real-world constraint: several athletes may decline testing or treatment owing to inconvenience, stigma, privacy concerns, or perceived performance implications. Teams should therefore embed shared decision-making, clear communication about confidentiality and risk/benefit trade-offs, and practical support for adherence within their pathways. Once diagnosed, OSA is often treatable, but treatment choice is personalised (e.g., on the basis of severity, symptoms, anatomy, comorbidities, and athlete preference); options can include positive airway pressure therapy (PAP), mandibular advancement devices (MAD) and positional therapy [1]. Behavioral sleep interventions remain important but should be integrated with, not substituted for, the treatment of underlying sleep-disordered breathing. For concussed athletes, incorporating OSA screening and, where appropriate, treatment into concussion management pathways may offer a modifiable target to support recovery, while acknowledging that direct interventional evidence in athlete concussion cohorts is still needed. Evidence for OSA in athletes remains limited by small sample sizes, male-dominated cohorts, heterogeneous diagnostic methods, and potential selection bias toward symptomatic athletes and those with higher body mass. Much of the objective evidence base to date comes from contact and collision sports; whether similar prevalence and consequences exist in endurance, aesthetic/weight-category, and female athlete cohorts remains uncertain. Prospective studies in female, endurance, and Paralympic athletes, as well as interventional trials testing whether treating OSA improves both health and performance outcomes, are urgently needed. This is particularly relevant for Paralympic athletes and disability populations in which sleep-disordered breathing risk may be elevated (e.g., spinal cord injury, neuromuscular disorders, ventilatory impairment), yet sport-specific screening and care pathways are underdeveloped. Meanwhile, OSA is unlikely to be the sole explanation for poor sleep quality or insomnia in athletes; rather, it should be considered alongside behavioral sleep restriction, circadian disruption, and insomnia as part of a structured differential diagnosis. For sports medicine to optimize performance and safeguard long-term wellbeing, recognizing and appropriately assessing obstructive sleep apnea in at-risk athlete groups should be a priority.
